# Short-Term Ambient Air Pollution and Urticaria in Guangzhou, China: Estimating the Association and Population Attributable Fraction

**DOI:** 10.3390/toxics11120949

**Published:** 2023-11-21

**Authors:** Huanli Wang, Morgan Matusik, Robert Wunderlich, Sarah E. Hanson, Kelly Babich, Lilianne Samad, Aaron M. Qian, Stephen Edward McMillin, Xingdong Ye, Sanquan Zhang, Yumei Liu, Xiaoyin Chen, Zhenjie Li, Hualiang Lin, Huilan Zhu, Xiaojie Wang

**Affiliations:** 1 Department of Dermatology, Guangzhou Institute of Dermatology, Guangzhou 510095, China; 2 Institute of Dermatology, Guangzhou Medical University, Guangzhou 510095, China; 3 University of New Mexico Hospital, Albuquerque, NM 87106, USA; 4 Mercy Health-West Hospital, Cincinnati, OH 45211, USA; 5 Bureau of Communicable Disease Control and Prevention, Missouri Department of Health and Senior Services, Jefferson City, MO 63103, USA; 6 Connecticut Department of Public Health, Office of Public Health Preparedness and Response, Hartford, CT 06134, USA; 7 Department of Epidemiology and Biostatistics, College for Public Health and Social Justice, Saint Louis University, Saint Louis, MO 63104, USA; 8 Department of Psychology, College of Arts and Sciences, Saint Louis University, Saint Louis, MO 63108, USA; 9 School of Social Work, Saint Louis University, Saint Louis, MO 63103, USA; 10 Department of Epidemiology, School of Public Health, Sun Yat-sen University, Guangzhou 510080, China

**Keywords:** air pollution, urticaria, short-term, China, population attributable fraction

## Abstract

Limited evidence is available regarding the association between acute exposure to ambient air pollutants and the risk of urticaria, even though the skin is an organ with direct contact with the external environment. This study utilized generalized additive models to investigate the association between particulate matter with an aerodynamic diameter smaller than 10 μm (PM_10_) and 2.5 μm (PM_2.5_), nitrogen dioxide (NO_2_) and sulfur dioxide (SO_2_), and daily outpatient visits for urticaria in Guangzhou, China from 2013 to 2017. We also estimated the attributable fraction of urticaria outpatient visits due to air pollution. A total of 216,648 outpatient visits due to urticaria occurred during the study period. All air pollutants were significantly associated with an increased excess risk of urticaria. Each 10 μg/m^3^ increase in PM_2.5_, PM_10_, NO_2_, and SO_2_ was associated with an increase of 1.23% (95% CI: 0.42%, 2.06%), 0.88% (95% CI: 0.28%, 1.49%), 3.09% (95% CI: 2.16%, 4.03%), and 2.82% (95% CI: 0.93%, 4.74%) in hospital visits for urticaria at lag05, respectively. It was estimated that 3.77% (95% CI: 1.26%, 6.38%), 1.91% (95% CI: 0.60%, 3.26%), 6.36% (95% CI: 4.38%, 8.41%), and 0.08% (95% CI: 0.03%, 0.14%) of urticaria outpatient visits were attributable to PM_2.5_, PM_10_, NO_2_, and SO_2_ using the World Health Organization’s air quality guideline as the reference. Relatively stronger associations were observed during the cold season. This study indicates that short-term air pollution may play a significant role in outpatient visits for urticaria, and that such relationships could be modified by season.

## 1. Introduction

Urticaria, commonly known as hives, can stem from many different factors. It is a medical condition characterized by a raised, red, and often itchy rash on the outer layer of the skin. Symptoms of this condition are known to be triggered by certain foods, alcohol consumption, sunlight, medications, bacterial infections, pet dander, and pollen, among other causes [1]. Urticaria may be acute, presenting itself for a short period and often resolving quickly, or it can be chronically recurring, in which it presents itself over long periods or in sudden, frequent instances [2]. Although not typically life-threatening, the condition has the ability to cause immense discomfort and disruption to a patient’s daily life and personal appearance. Since urticaria often occurs as an allergic reaction to environmental triggers, the emergence of symptoms can be quite common in large, populated cities with high levels of air pollution [3].

Common urban pollutants, such as particulate matter with an aerodynamic diameter smaller than 10 μm (PM_10_) and 2.5 μm (PM_2.5_), nitrogen dioxide (NO_2_) and sulfur dioxide (SO_2_), have been shown by studies conducted in China and elsewhere to have significant and negative impacts on human health [4,5,6,7]. For example, one recently published study found that long-term ambient air pollution exposure was associated with incident asthma, subsequent cardiovascular disease, and death [7]. Another study conducted using data from the UK Biobank concluded that ambient air pollution was associated with the incidence and progression trajectory of chronic lung diseases [6]. Additionally, one time-stratified case-crossover study conducted in China provided consistent evidence that 10 μg/m^3^ increases in PM_2.5_, PM_10_, and NO_2_ were significantly associated with 1.43%, 1.06%, and 2.80% increases in risk of dementia mortality, corresponding to excess mortality rates of 4.87%, 5.50%, and 6.43%, respectively [8]. Though a large majority of the previous studies have mainly focused on the respiratory and cardiovascular effects of air pollution [9,10], air pollutants can have detrimental effects on all organ systems, including the integumentary system [11]. In one study, the results demonstrated an association between air pollution concentrations and various skin conditions, including acne, psoriasis, skin cancer, atopic dermatitis, eczema and urticaria [12,13,14]. However, limited evidence exists on the association between air pollution and skin conditions, with conflicting results regarding this association [14,15,16,17]. For instance, a time-series study revealed that SO_2_ and NO_2_ were significantly and positively associated with emergency room visits for urticaria, while no such association was observed between short-term exposure to particulate matter (both PM_2.5_ and PM_10_) and urticaria [14]. Another study designed via time stratification did not find a statistically significant association between ambient air pollution and the incidence of urticaria [17].

Thus, we conducted this study to quantitatively estimate the association between air pollutants and urticaria outpatient visits, taking into account meteorological factors, seasonal factors, and holiday patterns in Guangzhou, China. The findings may provide important insights into the etiology of related diseases and inform the implementation of effective air pollution control measures.

## 2. Methods

### 2.1. Study Location and Data Collection

This study was conducted in Guangzhou, China, a city with a population of 12.64 million people and a humid subtropical monsoon climate [18]. This study focused on residents living in Guangzhou for two main reasons. First, air monitoring stations are situated in close proximity to these living areas, which reduces the likelihood of exposure measurement errors. Secondly, Guangzhou, as a capital city with a developed economy, has a higher quality of health outcome data [19].

Information about urticaria outpatient admissions and disease diagnosis was obtained using electronic medical records from the Guangzhou Municipal Clinical Center for Dermatology (GMCCD) between January 2013 and December 2017. GMCCD is the only municipal hospital within the Guangzhou region that specializes in the treatment of skin conditions. The clinic outpatient data records included demographic information and clinical diagnoses performed using International Classification of Diseases, 10th revision (ICD-10) codes. While the records included all patients, the final participants in this study were limited to outpatient visitors diagnosed with urticaria, otherwise indicated with an ICD-10 code of L50 within allergy due to unspecified causes.

### 2.2. Air Pollution and Meteorological Data

Ambient air pollution data were obtained from the national air monitoring system. For the purposes of this study, daily air pollution data were sourced from 11 air monitoring stations in Guangzhou. The mean of daily concentrations from the 11 monitoring stations of each pollutant (PM_2.5_, PM_10_, NO_2_, and SO_2_) was used in the analysis. Weather information, including relative humidity and temperature, came from the National Weather Data Sharing System (http://data.cma.cn/) (accessed on 9 November 2023).

### 2.3. Statistical Analysis

Since hospital visits for urticaria are rare events, the data usually follow a Poisson distribution, leading to the development of a generalized additive model (GAM) with a quasi-Poisson link to predict the relationship between urticaria and air pollution [20]. Unpenalized cubic regression splines were used to account for non-linear trends in temporality, mean temperature, and relative humidity, while the day of the week and the presence of a public holiday were included as covariates with linear coefficients [21]. Degrees of freedom (df) were employed to govern the complexity of the smooth functions utilized for modeling relationships between predictors and the response variable in our analysis [22]. We established the df settings in accordance with the previous literature [20], using 6 df/year for both temporal trends and temperature and 3 df/year for relative humidity. The formula can be specified as follows:

log[E(Y*_t_*)] = α + β_1_ × *Z_t_* + s(*t*, df = 6/year) + s(Temp03, df = 6) + s(RH, df = 3) + β_2_ × DOW + β_3_ × PH (1)

where *t* represents the day of observation, E(Y*_t_*) is the expected number of urticaria hospital visits on day *t*, α represents the intercept, β_1_, β_2_, and β_3_ are corresponding regression coefficients, and *Z_t_* is the pollutant concentration on day *t*. For this analysis, the pollutants of interest are PM_2.5_, PM_10_, NO_2_, and SO_2_. s() is the unpenalized cubic spline function, Temp03 is the moving average of the daily mean temperature for the day of the urticaria visit and the three days prior, and DOW is a dummy variable denoting the day of the week for observation *t*. PH represents a binary variable indicating whether a public holiday occurred on day *t*, and RH represents the daily average relative humidity.

To explore the lagged associations, we constructed models with various lag structures, ranging from the same day (lag0, representing current-day air pollution exposure) to 5 days prior (lag5, corresponding to air pollution exposure from the previous 5 days). Additionally, we incorporated multi-day lags, which include the moving average of air pollutant concentrations from the previous day (lag01), the previous three days (lag03), and the previous 5 days (lag05) [23].

### 2.4. Stratified Analysis

To examine possible effect modification by seasons, data were stratified into cold and warm seasons. The cold season was defined as 1 October through 31 March, while the warm season was defined as April 1 through September 30. We tested the statistical significance of differences in the effect estimates between different seasons by calculating the 95% confidence interval (CI) using the following formula [20]:

(2) $$Q_{1}-Q_{2}\pm 1.96\sqrt{{{(SE}_{1})}^{2}+{\left({SE}_{2}\right)}^{2}}$$

where both *Q*_1_ and *Q*_2_ are the coefficients for each stratum (cold and warm), and SE_1_ and SE_2_ are their corresponding standard errors.

### 2.5. Estimating Attributable Burden of Urticaria Due to Air Pollution

Attributable number (AN) and population attributable fractions (PAF) were calculated as metrics to examine the burden of urticaria outpatient visits attributable to ambient pollution. The guideline values set by the WHO’s Air Quality Guidelines were used as the reference [24]. Specifically, the references for PM_2.5_, PM_10_, NO_2_, and SO_2_ were 15 μg/m^3^, 45 μg/m^3^, 25 μg/m^3^, and 40 μg/m^3^, respectively. The details about calculating AN and AF have been described elsewhere [25,26].

### 2.6. Sensitivity Analyses

Several sensitivity analyses were applied. First, both the df of the calendar time (5–8 df per year) and the df of meteorological factors (3–5 df) were changed. Second, analyses with various two-pollutant models were conducted.

We conducted all analyses using R software (Version 4.0.5) in this study. We considered *p*-values of less than 0.05 as indicative of statistical significance. Our results are reported as excess risks (ERs), equal to relative risk (RR)—1 × 100%. Specifically, we initially employed GAMs through the ‘mgcv’ package in R, enabling the estimation of coefficients (betas) related to various air pollutants. Subsequently, we extracted these estimated coefficients and applied the ‘exp’ function to exponentiate the differences between coefficients, effectively converting them into RRs. By subtracting 1 from RR and then multiplying by 100%, we obtained ER expressed as a percentage. The use of ER allowed us to express the magnitude of the risk increase in a more interpretable way.

## 3. Results

A total of 216,648 urticaria outpatient visits occurred in the study area during the study period. Table 1 shows the descriptive statistics of air pollutants, urticaria outpatient visits, and meteorological factors. On average, there were 119.8 outpatient visits per day due to urticaria. Daily concentrations of PM_2.5_, PM_10_, NO_2_, O_3_, and SO_2_ were 41.6, 62.3, 46.7, 52.9, and 15.6 μg/m^3^, respectively, during 2013–2017. The mean temperature was 22.0 °C and the relative humidity was 80.2%. Table 2 presents the Spearman correlation coefficients between the daily concentrations of air pollutants and meteorological factors. Notably, PM_2.5_ was strongly correlated with PM_10_ (r = 0.94). Generally, both PM_2.5_ and PM_10_ displayed moderate to high correlations with gaseous pollutants (r ranging from 0.37 to 0.75).

**Figure 1:**
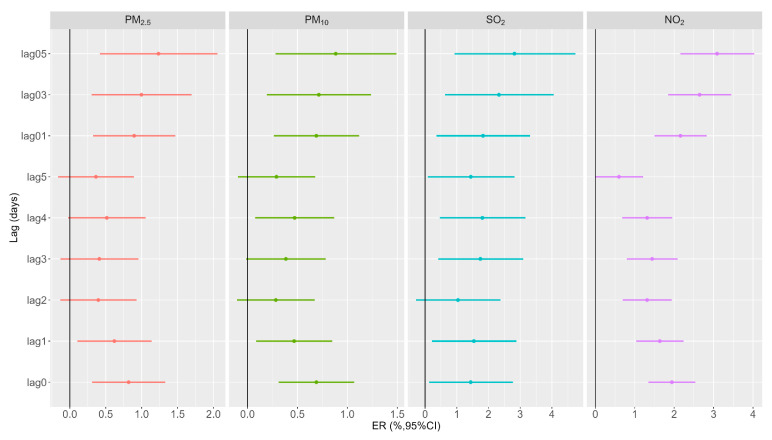
Excess risk (and 95% confidence interval) of urticaria outpatient visits for each 10 μg/m^3^ increment in PM_2.5_, PM_10_, SO_2_, and NO_2_ with different lag days.

Figure 1 demonstrates the excess risk values of daily urticaria outpatient visits associated with 10 μg/m^3^ increases in PM_2.5_, PM_10_, SO_2_, and NO_2_, after adjustment for relative humidity, day of the week, presence of a public holiday, and the three-day moving average of temperature. Similar patterns of lagged effects were observed for different air pollutants. It was observed that all associations decreased from lag0 to lag5 in single-day lagged associations. The effects increased from lag01 to lag05, with the strongest effects at lag05. The multi-day lag effects of air pollutants were more strongly associated with urticaria outpatient visits as compared to any single lag day.

Specifically, PM_2.5_ showed a 0.82% (95% CI: 0.31%, 1.33%) increase in urticaria outpatient visits for every 10 μg/m^3^ increase at lag0. Notably, the cumulative effect of PM_2.5_ at lag05 (ER = 1.23%, 95% CI: 0.42%, 2.06%) displayed a stronger association with urticaria outpatient visits compared to any single lag day. For PM_10_, each 10 μg/m^3^ increase was associated with a 0.69% (95% CI: 0.31%, 1.07%) increase in urticaria outpatient visits at lag0. A similar association was observed at lag1, lag3, and lag4, although the effects of lag2 and lag5 did not exhibit a significant association. Multi-day lags, particularly lag05, revealed stronger associations (ER = 0.88%, 95% CI: 0.28%, 1.49%) with urticaria outpatient visits. Concerning SO_2_, a 10 μg/m^3^ increase was associated with a 1.44% (95% CI: 0.12%, 2.77%) increase in urticaria outpatient visits at lag0. Similar associations were noted at lag1, lag3, lag4, and lag5, although lag2 did not show a statistically significant association. The multi-day lag effects of SO_2_ also exhibited significance, with the highest increase observed at lag05 (ER = 2.82%, 95% CI: 0.93%, 4.74%) in urticaria outpatient visits. As for NO_2_, each 10 μg/m^3^ increase was linked to a 1.94% (95% CI: 1.35%, 2.54%) increase in urticaria hospital visits at lag0. Significant associations were also observed for lag1 through lag4. A cumulative effect was also observed, with the highest increase in urticaria hospital visits occurring at lag05 (ER = 3.09%, 95% CI: 2.16%, 4.03%).

**Figure 2:**
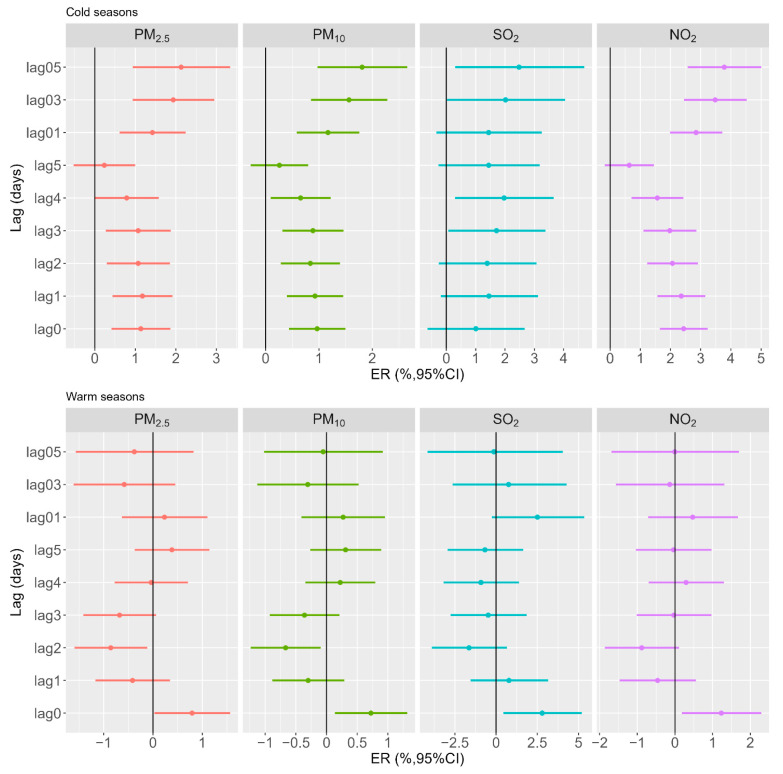
Excess risk (and 95% confidence interval) of urticaria outpatient visits for each 10 μg/m^3^ increment in PM_2.5_, PM_10_, SO_2_, and NO_2_ with different lag days stratified by seasons.

Figure 2 illustrates the ER with a 95% CI of urticaria outpatient visits per 10 μg/m^3^ increase in PM_2.5_, PM_10_, SO_2_, and NO_2_ with different lag days stratified by seasons. A statistically significant difference in the effects was observed between the two seasons. During the cold season, we found a consistent pattern in the effects of PM_2.5_, PM_10_, and NO_2_ on urticaria outpatient visits. The associations exhibited a slight decrease from lag0 to lag5, followed by a slight increase from lag01 to lag05, with the highest estimates observed at lag05. However, significant associations between air pollution exposure and urticaria outpatient visits were not observed during the warm season, except at lag0. In general, the estimates for the impact of various air pollutants on urticaria outpatient visits were higher during the cold seasons compared to the warm seasons. For example, during the cold season, we estimated increases of 2.13% (95% CI: 0.93%, 3.34%), 1.81% (95% CI: 0.97%, 2.65%), 2.48% (95% CI: 0.30%, 4.70%), and 3.78% (95% CI: 2.57%, 5.00%) in urticaria outpatient visits for each 10 μg/m^3^ increase in PM_2.5_, PM_10_, SO_2_, and NO_2_ at lag05. In contrast, during warm seasons, the estimated effects were −0.38% (95% CI: −1.56%, 0.82%), −0.06% (95% CI: −1.02%, 0.92%), 0.13% (95% CI: −4.14%, 4.04%), and −0.01% (95% CI: −1.68%, 1.70%).

Table 3 shows AN and PAF of urticaria outpatient visits due to ambient air pollution using WHO air quality guidelines. It was estimated that 3.77% (95% CI: 1.26%, 6.28%), 1.91% (95% CI: 0.60%, 3.26%), 6.36% (95% CI: 4.38%, 8.41%), and 0.08% (95% CI: 0.03%, 0.14%) of urticaria outpatient visits were attributable to PM_2.5_, PM_10_, NO_2_, and SO_2_, respectively. These estimates corresponded to 8174 (95% CI: 2735, 13825), 4130 (95% CI: 1290, 7066), 13,768 (95% CI: 9481, 18214), and 171 (95% CI: 54, 300) urticaria outpatient visits. A difference in the disease burden was found between pollutants, with the largest PAF and AN caused by NO_2_.

Sensitivity analyses suggested that the main findings were stable. For example, the effects remained largely consistent when the df was changed. For the effects of PM_2.5_, the corresponding ERs were 1.28% (95% CI: 0.46%, 2.10%) when altering the df of temporal trends from 6 to 5 (Table S1). In two-pollutant models, the estimates slightly decreased, but remained positive and significant (Table S2).

## 4. Discussion

This study is the first attempt to examine the effects of PM_2.5_, PM_10_, NO_2_, and SO_2_ on the risk of urticaria outpatient visits in Guangzhou, China. Findings conclude that short-term exposure to air pollutants was associated with increased urticaria outpatient visits. Additionally, the association was more prominent in cold seasons.

Findings were consistent with those of prior studies in Windsor, Canada; Beijing, China; and the San Joaquin Valley, California [14,15,16], while a contrary study conducted in Lanzhou, China found that the association was more pronounced during the warm season [27]. The geographical difference between the cities may contribute to the disparity in results. Guangzhou is a large maritime city in a subtropical climate, while Lanzhou is an inland city with a much smaller population and a more temperate climate. Additionally, a study of children in Singapore found no statistically significant association between air pollution and urticaria outpatient visits [17]. With the high prevalence of urticaria among children, it is possible that other common urticaria triggers outweighed the effects of air pollution among this population.

By stratifying for seasons, this study highlights the difference in the risk of daily urticaria due to exposure to air pollutants between cold and warm seasons. Air pollution had stronger effects on the frequency of urticaria outpatient visits during the cold seasons compared to the warm season. More research is needed to explore the reasons behind these relationships, but it is possible that the typical worsening of air pollution in the warm season means citizens are more conscious of the dangers and are more likely to take precautions to decrease exposure. It is also possible that Guangzhou’s subtropical climate leads to fewer people venturing outside in the warm season, while the cold season is more conducive to outdoor activity, leading to increased exposure to air pollution [28]. The average temperature highs in Guangzhou during the warm season are well above 25 or even 30 degrees Celsius, while the average temperature highs during the cold season are at a much more comfortable 20–25 degrees Celsius. Furthermore, the subtropical climate leads to more vegetative matter blooming during the cold season, increasing the number of allergens present in the air. These increased allergens may also contribute to the greater association between air pollution and urticaria outpatient visits during the cold season.

Another important finding of this study was the burden of urticaria outpatient visits attributable to air pollution. Substantial urticaria outpatient visits could have been avoided if the WHO air quality guidelines were adopted. These findings emphasize the need for stringent air quality regulations to protect the skin health of the population. A difference was observed in the attributable burdens of different pollutants, with the largest AN and AF caused by NO_2_. These findings can be instrumental in advocating for stronger environmental protection policies, as the lack of these policies may increase both poor health outcomes as well as healthcare costs through avoidable clinic visits.

The findings of this study have profound implications for both clinical practice and public health strategies focused on urticaria prevention. Our results highlight the pivotal and modifiable role of short-term exposure to ambient air pollution in the increased number of urticaria outpatient visits. From a clinical perspective, these findings underscore the urgency of developing evidence-based clinical guidelines to address urticaria associated with air pollution exposure. Clinicians should recommend preventive measures to their patients, such as using face masks and air purifiers, or limiting outdoor activities during high-level air pollution periods, especially during the cold season. From a public health perspective, our results provide a compelling rationale for governments and regulatory agencies to enact more stringent air quality standards and proactively reduce pollution levels. These initiatives hold the potential to reduce hospital visits related to urticaria and enhance the overall health and well-being of the population.

Though this study raises the possibility that air pollution increases outpatient visits for urticaria, there are several limitations to consider. As an ecological study, we acknowledge its inherent limitations in controlling for unmeasured potential confounders at the individual level due to the scarcity of available data. For example, critical variables such as clinical history and the presence of food and drug allergies were regrettably absent from the dataset. Additionally, considering urticaria’s resemblance to other dermatological conditions, like atopic dermatitis or eczema, misclassification may also have contributed to an underestimation of the true association.

## 5. Conclusions

Short-term exposure to ambient air pollution, including PM_2.5_, PM_10_, NO_2_, and SO_2_, can potentially have adverse effects on outpatient visits for urticaria in Guangzhou, China. These effects are influenced by the season, with more pronounced effects observed during the cold season. It is crucial to acknowledge that the generalizability of our findings is confined to regions sharing similar geographical attributes, temperature fluctuations, and disease prevalence. Therefore, careful attention should be paid to interpreting the results and further investigation is warranted. For residents of Guangzhou, our study underscores the importance of minimizing exposure to high-level air pollution, especially during the cold season, as a precautionary measure to reduce the risk of developing urticaria. In terms of policymaking, our research highlights the critical importance of implementing robust environmental policies aimed at curbing air pollution.

## Figures and Tables

**Table 1:** Air pollution, urticaria hospital visits, and meteorological factors in Guangzhou, China between 18 January 2013 and 31 December 2017.

	Daily Mean (SD)	Quantiles				
		Min	P25	P50	P75	Max
Pollutants, μg/m^3^						
PM_2.5_	41.6 (23.2)	6.5	24.2	35.8	53.1	156.4
PM_10_	62.3 (29.9)	10.8	39.6	55.1	78.2	208.7
NO_2_	46.7 (19.3)	4.4	33.2	42.4	56.0	155.4
O_3_	52.9 (29.9)	5.1	31.7	48.2	68.0	294.6
SO_2_	15.6 (9.6)	4.1	10.3	13.6	18.4	166.4
Meteorological factors						
Temperature (°C)	22.0 (6.1)	3.5	17.0	23.3	27.3	32.3
Relative Humidity (%)	80.2 (11.0)	31.0	74.0	81.0	88.0	100.0
Urticaria hospital visits, n	119.8 (36.9)	0.0	96.0	120.0	148.0	217.0

**Table 2:** Spearman correlation coefficients between the daily concentrations of air pollutants and meteorological factors.

	PM_2.5_	PM_10_	O_3_	SO_2_	NO_2_	Temperature	Relative Humidity
PM_2.5_	1.00	-	-	-	-	-	
PM_10_	0.94	1.00	-	-	-	-	
O_3_	0.37	0.42	1.00	-	-	-	
SO_2_	0.67	0.66	0.35	1.00	-	-	
NO_2_	0.70	0.75	0.07	0.49	1.00	-	
Temperature	−0.35	−0.25	0.21	−0.03	−0.33	1.00	
Relative Humidity	−0.26	−0.30	−0.47	−0.20	0.05	0.19	1.00

**Table 3:** The attributable numbers and fractions with 95% confidence intervals of urticaria hospital visits due to PM_2.5_, PM_10_, NO_2_, and SO_2_ concentrations at lag05 exceeding WHO air quality standard.

Pollutants	Attributable Fraction (%)	Attributable Number
PM_2.5_	3.77 (1.26, 6.38)	8174 (2735, 13,825)
PM_10_	1.91 (0.60, 3.26)	4130 (1290, 7066)
NO_2_	6.36 (4.38, 8.41)	13,768 (9481, 18,214)
SO_2_	0.08 (0.03, 0.14)	171 (54, 300)

The reference for PM_2.5_, PM_10_, NO_2_, and SO_2_ concentration was based on the World Health Organization’s Ambient Air Quality guidelines (15 μg/m^3^, 45 μg/m^3^, 25 μg/m^3^, and 40 μg/m^3^).

## Data Availability

Publicly available datasets were analyzed in this study. This data can be found here: https://www.ukbiobank.ac.uk/ (accessed on 16 October 2023).

## Supplementary Materials

The following supporting information can be downloaded at: https://www.mdpi.com/article/10.3390/toxics11120949/s1, Table S1: Sensitivity analysis for urticaria outpatient visits associated with each 10 μg/m^3^ increment of air pollution at lag05; Table S2: Excess risk and 95% confidence intervals of urticaria outpatient visits for each 10 μg/m^3^ increment in air pollution at lag05 in single and two-pollutant models in Guangzhou.
