# Supplementary material for: Short-Term Ambient Air Pollution and Urticaria in Guangzhou, China: Estimating the Association and Population Attributable Fraction
Source: Toxics. 2023 Nov 21;11(12):949. doi: 10.3390/toxics11120949 (PMC10747676; doi:10.3390/toxics11120949)
Supplement: Supplementary file 1 [file toxics-11-00949-s001.zip › toxics-2695432-supplementary.pdf]

Table S1. Sensitivity analysis for urticaria outpatient visits associated with each 10 μg/m^3 increment of air pollution at lag05.

Pollutants	Models	Urticaria
$PM_{2.5}$		
	df=5 for temporal trends	1.28 (0.46, 2.10)
	df=7 for temporal trends	1.01 (0.16, 1.87)
	df=8 for temporal trends	1.05 (0.19, 1.92)
	df=5 for temperature	1.24 (0.42, 2.06)
	df=7 for temperature	1.23 (0.42, 2.05)
	df=8 for temperature	1.23 (0.42, 2.05)
PM_{10}		
	df=5 for temporal trends	1.05 (0.47, 1.64)
	df=7 for temporal trends	0.94 (0.33, 1.56)
	df=8 for temporal trends	0.91 (0.26, 1.56)
	df=5 for temperature	0.89 (0.29, 1.50)
	df=7 for temperature	0.88 (0.28, 1.49)
	df=8 for temperature	0.88 (0.28, 1.49)
NO_2		
	df=5 for temporal trends	3.13 (2.24, 4.02)
	df=7 for temporal trends	2.81 (1.81, 3.82)
	df=8 for temporal trends	2.78 (1.79, 3.78)

	df=5 for temperature	3.10 (2.16, 4.04)
	df=7 for temperature	3.08 (2.15, 4.02)
	df=8 for temperature	3.09 (2.16, 4.03)
SO_2		
	df=5 for temporal trends	2.61 (0.86, 4.39)
	df=7 for temporal trends	3.35 (1.37, 5.37)
	df=8 for temporal trends	2.94 (1.03, 4.90)
	df=5 for temperature	2.81 (0.92, 4.72)
	df=7 for temperature	2.82 (0.94, 4.74)
	df=8 for temperature	2.82 (0.94, 4.74)

Table S2. Excess risk and 95% confidence intervals of urticaria outpatient visits for each 10 μg/m^3 increment in air pollution at lag05 in single and two-pollutant models in Guangzhou.

Pollutants	Two-pollutant models	Urticaria
$PM_{2.5}$	Control for O_3	1.29 (0.43, 2.15)
	Control for SO_2	1.08 (0.21, 1.95)
PM_{10}	Control for O_3	0.93 (0.29, 1.57)
	Control for SO_2	0.76 (0.15, 1.38)
NO_2	Control for $PM_{2.5}$	3.10 (2.04, 4.16)
	Control for PM_{10}	3.05 (1.92, 4.18)
	Control for O_3	3.21 (2.24, 4.18)
SO_2	Control for $PM_{2.5}$	2.08 (0.11, 4.08)
	Control for PM_{10}	2.53 (0.58, 4.53)
	Control for O_3	2.80 (0.88, 4.76)
